# Chemical Bonds Formed in Solid Wood by Reaction with Maleic Anhydride and Sodium Hypophosphite

**DOI:** 10.3390/ma17194856

**Published:** 2024-10-02

**Authors:** Injeong Kim, Oleg N. Antzutkin, Faiz Ullah Shah, Olov Karlsson, Dennis Jones, Dick Sandberg

**Affiliations:** 1 Wood Science and Engineering, Luleå University of Technology, Forskargatan 1, 93177 Skellefteå, Sweden; olov.karlsson@ltu.se (O.K.); dennis.jones@ltu.se (D.J.); 2 Chemical Engineering, Luleå University of Technology, 97187 Luleå, Sweden; oleg.antzutkin@ltu.se (O.N.A.); faiz.ullah@ltu.se (F.U.S.); 3 Biotechnical Faculty, University of Ljubljana, Rožna Dolina, Cesta VIII/34, 1000 Ljubljana, Slovenia; 4 InnoRenew CoE, Livade 6a, 6310 Izola, Slovenia; dick.sandberg@outlook.com; 5 Department of Manufacturing and Civil Engineering, Norwegian University of Science and Technology (NTNU), 2821 Gjøvik, Norway

**Keywords:** wood modification, maleic anhydride, sodium hypophosphite, ^13^C and ^31^P MAS NMR, XPS

## Abstract

The reaction of wood with maleic anhydride (MA) and sodium hypophosphite (SHP) has been identified as a viable modification method, with macroscopical properties indicating formation of cross-linking to explain the results. However, the chemical reaction between wood and the modification reagents has not been studied yet. To resolve this, the reaction was studied with solid-state ^13^C cross-polarization magic-angle-spinning (CP-MAS) and ^31^P MAS nuclear magnetic resonance (NMR) and X-ray photoelectron spectroscopy (XPS) to reveal the formation of bonds between wood components, MA and SHP during the treatments to explain the formation of cross-linking and the possible fixation of phosphorus in wood. XPS, solid state ^13^C and ^31^P MAS NMR revealed the maleation of wood in the absence of SHP, whilst its presence led to forming a succinic adduct observed through the C-P bond formation, as evidenced by the loss of the maleate C=C bonds at around 130 ppm and the upfield shift of the peak at 165–175 ppm, which was also significantly smoothed, as well as the increase in a peak at 26 ppm due to the reaction between the maleate group and SHP; however, the C-P-C bond could not be unambiguously rationalized from the obtained data. On the other hand, a resonance line at 16 ppm in ^31^P MAS NMR and the peaks in the XPS P 2p spectrum suggested the formation of a cross-linked structure at low concentrations of SHP, which was more likely to be phosphonate (C-P-O) than organophosphinic acid (C-P-C). The results herein provide a greater fundamental understanding of the mechanisms involved in the reaction of wood, MA and SHP, providing further scope for improved treatment systems in the future.

## 1. Introduction

Whilst wood protection with preservative methods is often carried out with the use of transition metals and synthetic fungicides, wood modification offers an alternative method for protecting wood from decay [1,2], avoiding the use of said chemicals. Modified wood is then intended to be non-toxic in service, and the disposal of this wood at the end of its life does not result in the generation of any toxic residues, i.e., can be disposed at the end of a product’s life cycle without presenting any environmental hazards greater than those that are associated with the disposal of unmodified wood. Maleic anhydride (MA) can be considered as a chemical suitable for wood modification. The main objective of modifying wood is to reduce interaction with water and moisture as a result of chemically altering the wood structure [3], thereby providing dimensional stability and, in turn, increased biological resistance. This effect involves the physical and chemical blocking of water interacting with the components of wood, thereby restricting conditions conducive to microorganisms becoming established [1,3,4]. Other methods also employ the possibility of cross-linking the structure to restrict the moisture-induced motion of fungi and enzymes produced by microorganisms [5,6,7].

Previous studies on the esterification of wood with MA showed that the ester bond formed between hydroxyl groups within wood and MA was affected by the more favorable competing hydrolysis reaction of MA with water at elevated temperatures; therefore, it required additional chemical agents to form stable cross-linkages [8,9,10,11]. In studies of cotton cellulose, it was suggested that sodium hypophosphite (SHP) may react with the unsaturated sites on cellulose maleate to form a cross-link, resulting in a material with enhanced wrinkle resistance and fire retardancy after laundry cycles [12,13], as well as an improved breaking strength and tearing strength [14]. A similar tendency was observed in wood modified with MA and SHP, whereby the modified wood showed improved dimensional stability and decreased affinity in the wood cell wall to water [11,15].

Our previous study [11] indicated a degree of cross-linking in wood treated with MA and low concentrations of SHP (but not by treatment of wood with only SHP) during repeated wet/dry cycle tests. On the other hand, for wood treated with MA and high concentrations of SHP, a considerable amount of mass from samples gained by modification treatments was reduced after consecutive wet–dry cyclic conditioning, resulting in a reduction in dimensional stability in the modified wood. The result with low concentrations indirectly supported that cross-linking had occurred, possibly involving a C-P-C-bonded structure between the modified wood components, whereas the one with high concentration did not, suggesting that the reaction between wood components, MA and SHP might be different from the reaction between cotton, maleic acid and SHP suggested by Wu and Yang [12]. The reason might be due to the chemical and structural differences between cotton cellulose and wood; while cotton cellulose is mostly constituted of cellulose with a high degree of polymerization and crystallinity, wood is composed of a mixture of cellulose, hemicelluloses and lignin in complex structures, resulting in a more amorphous material with a different steric hindrance compared to cotton. Hence, it is necessary to investigate the chemical bonds between wood, MA and SHP. This study will not only reveal the cross-linking system but also may suggest a new method of fixation in regard to phosphorus in wood.

Solid-state NMR spectroscopy (SS-NMR) has been applied in structural studies of wood and its main components, with this method being popular as it allows samples to be studied without the use of solvents. Whilst conventional proton (^1^H) NMR is the most commonly used method, other elements displaying nuclear spin states can also be measured, allowing for the elucidation of chemical structures. Within organic chemistry, ^13^C cross-polarization magic-angle-spinning NMR measurements (^13^C NMR in the liquid form and ^13^C CP-MAS SS-NMR in the solid-state form) have become an established spectroscopic method and have been applied to investigate changes in the chemical structure of wood as a result of modification [16,17,18,19]. In this study, the chemical structure of wood treated with MA and SHP was investigated with SS-NMR. By choosing the respective nuclei ^31^P and ^13^C for the NMR experiments, the identity of bonds between wood and the reagents could be studied in more detail and compared with data provided by studies with X-ray photoelectron spectroscopy (XPS). The objective of the study was to reveal the presence and structure of chemical bonds between maleated wood and SHP and to identify how this is influenced by the concentration of SHP reagent.

## 2. Materials and Methods

### 2.1. Materials

Industrially dried Scots pine (*Pinus sylvestris* L.) sapwood, a commercially important species in Sweden, known for its relative ease of impregnation, was purchased locally from XL Bygg (Skellefteå, Sweden). The initial moisture content of the wood was 7.5 ± 0.1%, and the oven-dried density was 534.6 ± 8.9 kg/m^3^. Maleic anhydride (MA) (CAS No. 108-31-6) for synthesis was purchased from Sigma–Aldrich (Saint Louis, MO, USA) and sodium hypophosphite monohydrate 98% (CAS No. 7681-53-0) was purchased from Alfa Aesar (Haverhill, MA, USA). Distilled water was purchased from Brenntag Nordic AB (Malmö, Sweden), and technical grade acetone was purchased from VWR Chemicals (Radnor, Brooklyn, NY, USA).

### 2.2. Preparation of Specimens

Four types of specimens were prepared: untreated wood, maleated (MS00), wood treated with MA and 0.5 M of SHP (MS05) and wood treated with MA and 3.5 M of SHP (MS35).

The specimens were modified with the concentrations of MA in acetone of 3.5 M and SHP in water of 0.5 M and 3.5 M. The concentration of solutions was based on the results of a previous study [11] which showed the best dimensional stability on repetitive wet–dry testing. The process was as follows: prior to the treatment, Scots pine sapwood was shaved into small thin slices, shorter than 0.5 cm, with a razor blade along the fiber direction. These samples were placed in a paper thimble, covered with mineral wool and placed in a Soxhlet apparatus. The extraction was performed for 6 h with acetone and water (4:1). The samples were dried in the oven at 103 °C until the equilibrium of weight was reached. The oven-dried samples were cooled down in a desiccator containing silica gel to retain their dryness.

The samples were soaked in a solution of MA in acetone (3.5 M) until all the samples sank to the bottom of the solution. Afterwards, the samples were wrapped with aluminum foil and heated in an oven at 115 °C for 2 h. The dried samples were then impregnated in aqueous solutions of SHP for 30 min at reduced pressure, followed by heating in oven at 170 °C for 6 h.

All specimens were treated with distilled water for 30 min under reduced pressure, followed by soaking under water for 24 h to remove excess chemical agents. Afterwards, all specimens were air-dried in a fume hood for a period of 24 h, followed by heating at 103 °C until equilibrium was achieved.

### 2.3. Analysis with Solid-State Nuclear Magnetic Resonance (SS-NMR)

Solid-state ^13^C cross-polarization (CP) magic-angle-spinning (MAS), ^13^C CP-MAS, and the direct excitation ^31^P MAS SS-NMR spectra, both with a high-power ^1^H-decoupling (spinal64, 90 kHz nutation frequency of protons), were obtained using “Varian” 5 mm MAS probes on a Avance-III 400 NMR spectrometer (Brucker, Billerica, USA) with a superconducting “zero-helium boil-off” Ascend Aeon magnet (B_0_ = 9.4 T). The ^13^C operating frequency was 100.64 MHz. The proton π/2 pulse duration in the ^13^C CP-MAS SS-NMR experiment was 3.3 μs, the CP mixing time was 2.0 ms and 4096 signal transients, spaced by a relaxation delay of 2.0 s, were accumulated.

^31^P MAS SS-NMR experiments were performed with a 162.01 MHz operating frequency, a ^31^P π/2 pulse duration of 1.4 μs, and 4000 signal transients, interspaced by relaxation delays of 30.0 s, were accumulated. The spinning frequency was 8000 ± 2 Hz in both the ^13^C CP-MAS and ^31^P MAS SS-NMR experiments.

### 2.4. Analysis with X-ray Photoelectron Spectroscopy (XPS)

The XPS spectra were collected with a Kratos Axis Ultra DLD electron spectrometer (Kratos Analytical, Manchester, UK) using a monochromated Al Kα source operated at 150 W. The analyzer pass energy of 160 eV for acquiring survey spectra and a pass energy of 20 eV for individual photoelectron lines were used. The surface potential was stabilized by the spectrometer charge neutralization system. The binding energy (BE) scale was referenced to the C 1s line of aliphatic carbon, set at 285.0 eV. Processing the spectra was accomplished with the Kratos software version 2. To avoid possible X-ray degradation effects, C 1s and O 1s spectra were acquired first within 10 min before the survey spectrum and spectra of minor elements.

## 3. Results and Discussion

**Figure 1:**
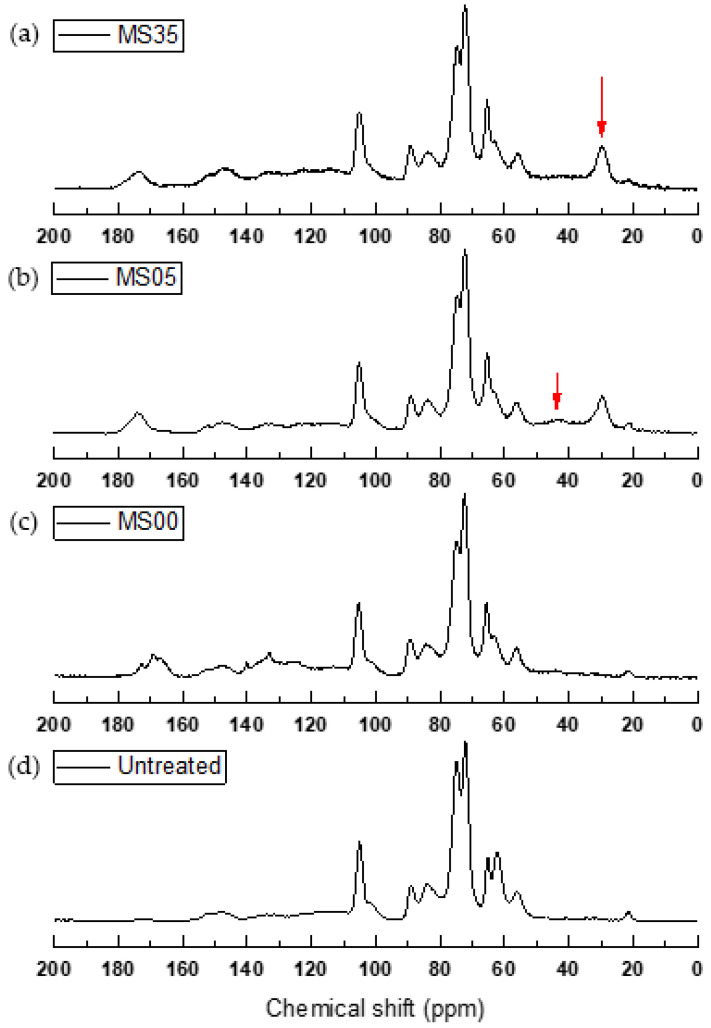
^13^C CP-MAS SS-NMR of Scots pine sapwood: (**a**) treated with maleic anhydride (MA) and 3.5 M sodium hypophosphite (SHP) (MS35); (**b**) treated with MA and 0.5 M SHP (MS05); (**c**) maleated; (**d**) untreated. The red arrow in (**a**) indicates the resonance at 26 ppm, whilst the arrow in (**b**) indicates the resonance at 43 ppm.

The chemical shifts observed in the ^13^C CP-MAS SS-NMR spectra of untreated, maleated wood (MS00) and wood modified with MA and SHP (MS05 and MS35) are shown in Figure 1. The reference sample (untreated) shows the carbons of cellulose (dominant) and hemicelluloses (C1 (cellulose: 105 ppm, hemicellulose: 102 ppm), C4 of cellulose (crystalline 89 ppm; amorphous 84 ppm), C2/C3/C5 of cellulose (75 and 72 ppm), and C6 of cellulose (crystalline 65 ppm; amorphous 62 ppm)) and lignin (methoxy group (56 ppm) quaternary olefinic or aromatic carbon (127–143 ppm), olefinic or aromatic carbon with hydroxyl or etherified substituents (143–167 ppm) and carbonyl groups (195–225 ppm) [18,20,21,22]).

Compared to untreated samples, all three treatments showed a decrease in C-2 carbohydrate resonance at 75 ppm, which might be due to the deacetylation of glucomannans or the opening of some pyranose rings during heat treatment [23]. Resonances around 102 ppm assigned to C-1 of hemicelluloses were reduced by the treatments (Figure 1). The resonances due to amorphous carbohydrates (62 ppm and 84 ppm) decreased by the treatment with MA, while the crystalline carbohydrates resonance lines (65 and 89 ppm) increased. This could be due to preferential esterification of amorphous carbohydrates by MA.

The cluster of resonance lines at 165–175 ppm in the maleated product (MS00) could involve formation of ester bonds to the wood matrix possibly involving at least one of the carboxylic groups in MA. As a comparison, only a small upfield shift of carboxyl group could be seen in ^13^C CP-MAS SS-NMR for maleic acid when esterified with methyl as well as ethyl groups [24]. Furthermore, the carbon–carbon double (C=C) bonds in maleic acid and the corresponding ester bonded maleic acid have resonance signals of around 120–140 ppm. As the signals at 120–140 ppm were significantly reduced by treatment with a low content of sodium hypophosphite (MS05), the double bond in maleate appeared to have been reduced by the addition of SHP. This is supported by the study of FTIR spectra of the maleated and SHP-treated product [11]. The wood modified with MA and SHP has an additional ^13^C chemical shift at 29 ppm, which is most likely attributed to the CH_2_ of the succinate derivate structure, produced by reduction in the C=C of wood maleate during the SHP treatment. An upfield shift and a smoother cluster of resonances at 165–175 ppm was seen by the treatment with SHP and was probably related to the formation of the succinate derivative. In the spectra of MS05, but not for MS35, an additional broad signal at 43 ppm was observed, which might be due to the formation of a carbon phosphorous (C-P) bond by a reduction in the carbon–carbon double bond in the maleated wood polymer [25].

**Figure 2:**
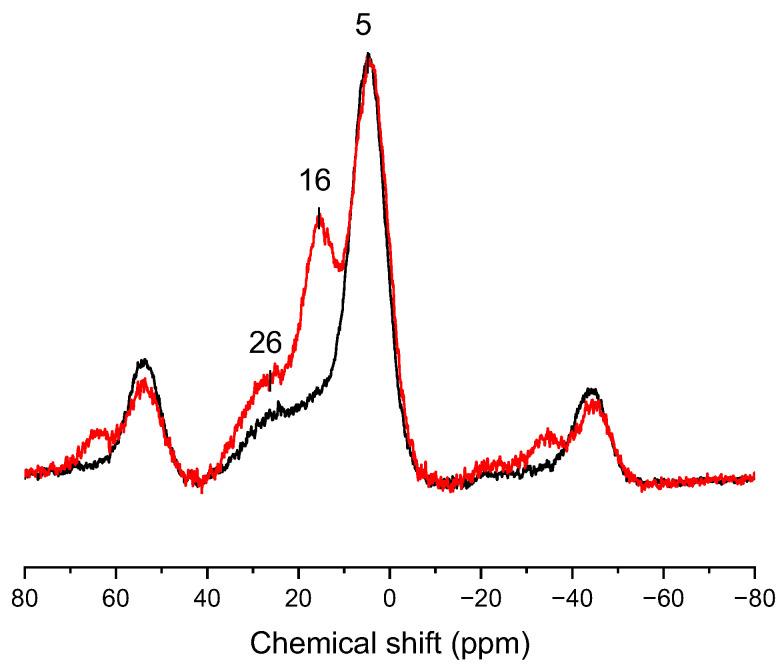
^31^P single-pulse MAS SS-NMR of wood maleate treated with 0.5 M SHP (MS05, red) and 3.5 M SHP (MS35, black).

Figure 2 shows the solid state direct excitation ^31^P MAS SS-NMR spectra of wood treated with MA and two different concentrations of SHP. Both specimens showed P-sites with chemical shifts at 5 ppm and 26 ppm. The resonance line at 5 ppm indicates the formation of phosphate monoester. In addition, the resonance at 26 ppm is likely to be due to the formation of phosphonate from reaction products between maleate and SHP. This is supported by a similar chemical shift being observed in other studies [25,26,27,28]. The specimen treated with 0.5 mol/L SHP clearly showed an additional signal at 16 ppm, which is likely to be a phosphonate ester [27,28]. The resonances from +50 to +70 ppm and from −20 to 50 ppm are (±1) spinning sidebands, respectively, from the main resonance signals.

**Figure 3:**
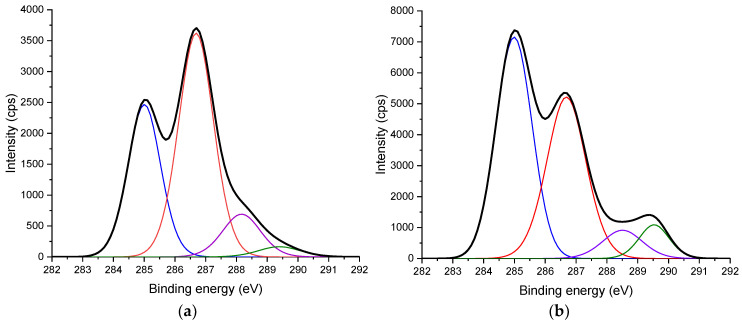
XPS C 1s spectrum of Scots pine sapwood: (**a**) untreated; (**b**) modified with 3.5 M MA and 0.5 M SHP. (approx. binding energy of C1 (blue): 285 eV, C2 (red): 287 eV, C3 (purple): 288.2 eV and C4 (green): 289.4 eV).

**Figure 4:**
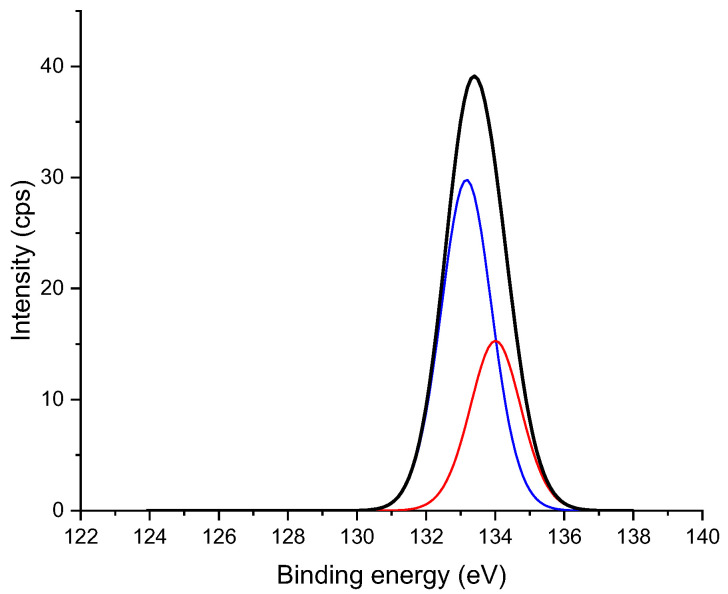
XPS P 2p spectrum of Scots pine sapwood modified with 3.5 M MA and 0.5 M SHP. The deconvoluted absorption located at 132.9 eV is C-P (blue) and the absorption located at 134 eV is O-P (red).

To further characterize the chemical structures of treated products, studies with X-ray techniques (XPS) were undertaken. MA with the formula of C_4_H_2_O_3_ could result in a decrease in the O/C ratio in the modified wood product. From the spectra, the atomic ratio of O/C could be calculated and was found to decrease after modification treatments (Table 1). Figure 3 shows the C 1s spectrum of specimens. Based on the study of Dorris and Gray [29], the C 1s absorptions were categorized in the following way: C1-carbon, which bonded to carbon or hydrogen (C-C, C=C, and C-H), C2-carbon, which bonded to single non-carbonyl oxygen (C-O and C-O-C), C3-carbon, which bonded to a carbonyl or two non-carbonyl oxygen (C=O and O-C-O), and C4-carbon, which corresponds to the O-C=O bonding of carboxylic derivatives. The proportion of C1 absorption and C4 absorption to the total carbon absorptions increased by the modification treatment while the proportion of the other two absorptions (C2 and C3) decreased. The increased intensity of the signal representing C1 and C4 could be due to increased amounts of hydrocarbons and the free and esterified carboxylic groups achieved by MA-modification of wood components. The deconvoluted absorptions at 132.9 and 134 eV of P 2p spectrum in Figure 4 can be assigned to the P-C and P-O bond in maleated wood treated with SHP, respectively [27,28].

In our previous study [11], the wood treated at a low concentration (0.5 M) of SHP showed enhanced dimensional stability with possible cross-linking, while the one treated with a high concentration (3.5 M) of SHP did not. Comparing the SS-NMR spectra of those two products, the chemical shift at 43 ppm in ^13^C SS-NMR and 16 ppm in ^31^P SS-NMR could be related to the cross-linking reaction with the lower amounts of SHP and maleated wood. However, unlike the hypothesis, which expected the formation of organophosphinic acid containing a C-P-C bond between two maleated wood polymer structures, the resonance line found at 16 ppm in ^31^P SS-NMR is more likely to belong to phosphonate, which is a C-P-O group.

The reaction between maleate and hypophosphite to form 2-(oxidohydrophosphoryl) succinate is known to occur under aqueous conditions [25]. When the aqueous solution of SHP was added to the wood maleate and heated, the additional reaction of hypophosphite to maleate could therefore occur. The resonances at 43 ppm in ^13^C SS-NMR and 26 ppm in ^31^P SS-NMR of modified samples are similar to those observed in the previous study [25]. However, it is unlikely that further cross-linking reactions, which could involve reactions of another maleate with the phosphorus atom in the formed H-phosphinate, have occurred. The literature evidence suggests that there are multiple examples of two reported approaches for the alkylation of H-phosphinate: one is a base-promoted H-phosphinate alkylation, the other is a silicon method which consists of silylating H-phosphinic acids, followed by an Arbuzov-like reaction with alkyl halides [30,31,32,33,34]. The hydrophosphinylation can occur either in a (transition)metal-catalyzed reaction or following a radical pathway [35,36]. The high temperature applied with addition of SHP (170 °C) could have been enough to overcome the P-H bonding energy of the PH_2_ moiety of SHP. However, the energy required for the release of the second hydrogen from phosphinate is higher than that of hypophosphite because the phosphinylidene moiety P(O)-H can tautomerize to form P-OH, which decreases P-H reactivity [36]. As the reaction in this study involves maleate and a salt-based catalyst, it is hard to assume that the alkylation of phosphinate took place as in previous studies on alkylation.

**Figure 5:**
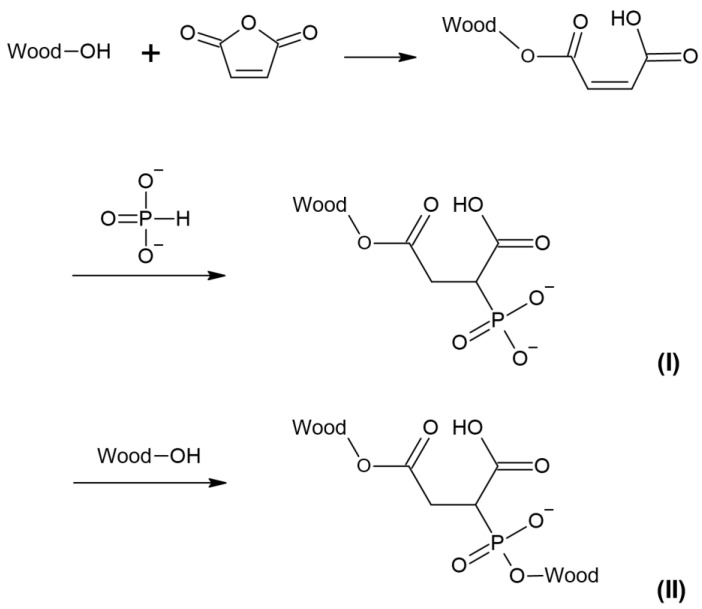
Tentative structure of cross-linking formed in MS05, formation of phosphonate (**I**) and formation of cross-linking (**II**).

The resonance line at 16 ppm is, however, not revealed in the ^31^P SS-NMR of the specimen treated with a higher concentration (3.5 M) of SHP, which indicated that alternative reactions might also exist. In our previous study, the dimensional stability of treated wood was achieved when the wood was treated with 3.5 M of MA and 0.5 M of SHP (MS05), but not when the wood was treated with 3.5 M of MA and 3.5 M of SHP (MS35) [11]. It is therefore logical to assume that the ^31^P SS-NMR line at 16 ppm is related to cross-linking. It had previously been reported that the phosphonic acid formed from SHP could react with the hydroxyl groups in lignin, resulting in covalent P-O-C bonding [37,38]. Other studies using ^31^P SS-NMR and XPS [27,28] had shown the presence of the phosphonate (I) as opposed to the 2-(oxidohydrophosphoryl) succinate, was more likely to result in cross-linking between the wood hydroxyls and the wood maleate structure (II), as postulated in Figure 5. It can also explain why the treatment at a low concentration of SHP was stable during repetitive wet–dry tests, since the phosphonates are known to be resistant to chemical hydrolysis and thermal decomposition [39,40,41,42].

The results herein, and those from earlier publications in this study [11,15], have focused on the properties of MA/SHP-treated solid wood. A comparative study using citric acid and SHP [43] suggested that, in terms of overall performance, there was no benefit from the use of SHP. The results from this extended study appear to show this is not the case with citric acid/SHP. Additional benefits may be afforded through the combination of MA/SHP and additional modifiers, such as itaconic acid [44], as suggested for potential protective coatings.

## 4. Conclusions

In this study, the presence and nature of chemical bonds between wood and chemical agents formed by modifying wood with MA and SHP was studied with XPS and solid-state ^13^C and ^31^P NMR techniques. The objective was to reveal the cross-linking formed between wood, MA and SHP and, in particular, how phosphorus has become fixed in wood.

A cross-linked structure formed in wood by modifying with MA and SHP is more likely to be of a phosphonate type of structure than an organophosphinic acid type involving a C-P-C bond. The dimensional stability of modified wood is proposed to be due to the formation of stable phosphonate links between wood and maleated wood components. The formation of stable phosphonate links shown in this study indicates a possibility of fixation of phosphorus in wood. Further studies on the properties of the modified wood, such as durability, are necessary to investigate the influence of the modification on wood properties for both solid wood and wood-based products. Since the presence of phosphorus is known to act as a fire retardant, further work will determine whether treatment with MA and SHP results in enhanced fire resistance.

## Figures and Tables

**Table 1:** Relative surface composition in atomic percentage (atomic %) and atomic ratio for untreated and modified Scots pine sapwood from analysis with XPS.

	Atom %				Atomic Ratio
	C	O	P	N	O/C
Untreated	68.5	31.1	-	0.5	0.45
MS05	72.3	27.3	0.2	0.2	0.38

## Data Availability

The original contributions presented in the study are included in the article, further inquiries can be directed to the corresponding author.

## References

[1] Hill C.A.S. (2006). Wood Modification—Chemical, Thermal and Other Processes. Wiley Series in Renewable Resources.

[2] Sandberg D., Kutnar A., Karlsson O., Jones D. (2021). Wood Modification Technologies: Principles, Sustainability, and the Need for Innovation.

[3] Thybring E.E., Fredriksson M. (2021). Wood modification as a tool to understand moisture in wood. Forests.

[4] Mai C., Militz H., Niemz P., Teischinger A., Sandberg D. (2023). Wood Modification. Springer Handbook of Wood Science and Technology.

[5] Yasuda R., Minato K. (1994). Chemical modification of wood by non-formaldehyde cross-linking reagents: Part 2. Moisture adsorption and creep properties. Wood Sci. Technol..

[6] Williams F.C., Hale M.D. (2003). The resistance of wood chemically modified with isocyanates: The role of moisture content in decay suppression. Int. Biodeterior. Biodegrad..

[7] Emmerich L., Bollmus S., Militz H. (2019). Wood modification with DMDHEU (1.3-dimethylol-4.5-dihydroxyethyleneurea)—State of the art, recent research activities and future perspectives. Wood Mater. Sci. Eng..

[8] Matsuda H., Ueda M., Murakami K. (1988). Oligoesterified woods based on anhydride and epoxide (I). Preparation and dimensional stability of oligoesterified woods by stepwise addition reactions. Mokuzai Gakkaishi.

[9] Iwamoto Y., Ito T. (2005). Vapor phase reaction of wood with maleic anhydride (I): Dimensional stability and durability of treated wood. J. Wood Sci..

[10] Essoua Essoua G.G., Blanchet P., Landry V., Beauregard R. (2015). Maleic anhydride treated wood: Effects of drying time and esterification temperature on properties. BioResources.

[11] Kim I., Karlsson O., Jones D., Mantanis G., Sandberg D. (2021). Dimensional stabilisation of Scots pine (*Pinus sylvestris* L.) sapwood by reaction with maleic anhydride and sodium hypophosphite. Eur. J. Wood Wood Prod..

[12] Wu X., Yang C.Q. (2008). Flame retardant finishing of cotton fleece fabric- part III: The combination of maleic acid and sodium hypophosphite. J. Fire Sci..

[13] Hameed S., Hussain M.A., Masood R., Haseeb M.T. (2016). Cross-linking of cotton fabric using maleic anhydride and sodium hypophosphite. Cellul. Chem. Technol..

[14] Peng H., Yang C.Q., Wang S. (2012). Nonformaldehyde durable press finishing of cotton fabrics using the combination of maleic acid and sodium hypophosphite. Carbohydr. Polym..

[15] Kim I., Thybring E.E., Karlsson O., Jones D., Mantanis G.I., Sandberg D. (2021). Characterisation of moisture in Scots pine (*Pinus sylvestris* L.) sapwood modified with maleic anhydride and sodium hypophosphite. Forests.

[16] Boonstra M.G., Pizzi A., Tekely P., Pendlebury J. (1996). Chemical Modification of Norway Spruce and Scots Pine. A ^13^C NMR CP-MAS Study of the Reactivity and Reactions of Polymeric Wood Components with Acetic Anhydride. Holzforschung.

[17] Chang S.-T., Chang H.-T. (2001). Comparisons of the photostability of esterified wood. Polym. Degrad. Stab..

[18] Wikberg H. (2004). Advanced Solid State NMR Spectroscopic Techniques in the Study of Thermally Modified Wood. Ph.D. Thesis.

[19] Martha R., Mubarok M., Batubara I., Rahayu I.S., Setiono L., Darmawan W., Akong F.O., George B., Gérardin C., Gérardin P. (2021). Errect of furfurylation treatment on technological properties of short rotation teak wood. J. Mater. Res. Technol..

[20] Gil A.M., Neto C.P. (1999). Solid-state NMR studies of wood and other lignocellulosic materials. Annu. Rep. NMR Spectrosc..

[21] Capanema E.A., Balakshin M.Y., Kadla J.F. (2004). A comprehensive approach for quantitative lignin characterization by NMR spectroscopy. J. Agric. Food Chem..

[22] Sievers C., Marzialetti T., Hoskins T.J., Olarte M.B.V., Agrawal P.K., Jones C.W. (2009). Quantitative solid state NMR analysis of residues from acid hydrolysis of loblolly pine wood. Bioresour. Technol..

[23] Tjeerdsma B.F., Boonstra M., Pizzi A., Tekely P., Militz H. (1998). Characterisation of thermally modified wood: Molecular reasons for wood performance improvement. Holz Roh-Werkst..

[24] Bonneaud C., Decostanzi M., Burgess J., Trusiano G., Burgess T., Bongiovanni R., Joly-Duhamel C., Friesen C.M. (2018). Synthesis of α,β-unsaturated esters of perfluoropolyalkylethers (PFPAEs) based on hexafluoropropylene oxide units for photopolymerization. RSC Adv..

[25] Huang Z., Chen Y., Kanan M.W. (2022). Hypophosphite addition to alkenes under solvent-free and non-acidic aqueous conditions. Chem. Commun..

[26] Harris R.K., Merwin L.H., Hägele G. (1989). Solid-state nuclear magnetic resonance study of a series of phosphonic and phosphinic acids. J. Chem. Soc. Faraday Trans..

[27] Puziy A.M., Poddubnaya O.I., Socha R.P., Gurgul J., Wisniewski M. (2008). XPS and NMR studies of phosphoric acid activated carbons. Carbon.

[28] Singh A.S., Advani J.H., Biradar A.V. (2020). Phosphonate functionalized carbon spheres as a Brønsted acid catalysts for valorization of bio-renewable α-pinene oxide to trans-carveol. Dalton Trans..

[29] Dorris G.M., Gray D.G. (1978). The surface analysis of paper and wood fibres by ESCA. II. Surface composition of mechanical pulps. Cellul. Chem. Technol..

[30] Baillie A.C., Cornell C.L., Wright B.J., Wright K. (1992). Synthesis of potential inhibitors of the enzyme pantothenate synthetase. Tetrahedron Lett..

[31] Baylis E.K. (1995). 1,1-Diethoxyethylphosphinates and phosphonites. Intermediates for the synthesis of functional phosphorus acids. Tetrahedron Lett..

[32] Froestl W., Mickel S.J., von Sprecher G., Diel P.J., Hall R.G., Maier L., Strub D., Melillo V., Baumann P.A., Bernasconi R. (1995). Phosphinic Acid Analogs of GABA. 1. New Potent and Selective GABAB Agonists. Med. Chem..

[33] Hall R.G., Kane P.D., Bittiger H., Froestl W. (1995). Phosphinic acid analogues of γ-aminobutyric acid (GABA). Synthesis of a new radioligand. J. Label. Compd. Radiopharm..

[34] Abrunhosa-Homas I., Sellers C.E., Montchamp J.-L. (2007). Alkylation of H-phosphinate esters under basic conditions. J. Org. Chem..

[35] Montchamp J.-L. (2014). Phosphinate chemistry in the 21st century: A viable alternative to the use of phosphorus trichloride in organophosphorus synthesis. Acc. Chem. Res..

[36] Lehrhofer L., Fliri L., Bacher M., Budischovwsky D., Sulaeva I., Hummel M., Rosenau T., Hettegger H. (2024). A mechanistic study on the alleged cellulose cross-linking system: Maleic acid/sodium hypophosphite. Carbohydr. Polym..

[37] Dorez G., Otazaghine B., Taguet A., Ferry L., Lopez-Cuesta J.M. (2014). Use of Py-GC/MS and PCFC to characterize the surface modification of flax fibres. J. Anal. Appl. Pyrolysis.

[38] Ferry L., Dorez G., Taguet A., Otazaghine B., Lopez-Cuesta J.M. (2015). Chemical modification of lignin by phosphorus molecules to improve the fire behavior of polybutylene succinate. Polym. Degrad. Stab..

[39] Gledhill W.E., Feijtel T.C., Hutzinger O.E. (1992). Environmental Properties and Safety Assessment of Organic Phosphonates Used for Detergent and Water Treatment Applications. The Handbook of Environmental Chemistry, Part F.

[40] Jaworska J., Van Genderen-Takken H., Hanstveit A., van de Plassche E., Feijtel T. (2002). Environmental risk assessment of phosphonates used in domestic laundry and cleaning agents in the Netherlands. Chemosphere.

[41] Nowack B. (2003). Environmental chemistry of phosphonates. Water Res..

[42] Lesueur C., Pfeffer M., Fuerhacker M. (2005). Photodegradation of phosphonates in water. Chemosphere.

[43] Feng X., Xiao Z., Sui S.-J., Wang Q., Xie Y. (2014). Esterification of wood with citric acid: The catalytic effects of sodium hypophosphite (SHP). Holzforschung.

[44] Ma W., Zhang Y., Li H. (2023). Synthesis and performance evaluation of carboxyl-rich low phosphorus copolymer scale inhibitor. J. App. Polym. Sci..

